# Improved motif-scaffolding with SE(3) flow matching

**Published:** 2024-01-08

**Authors:** Jason Yim, Andrew Campbell, Emile Mathieu, Andrew Y. K. Foong, Michael Gastegger, José Jiménez-Luna, Sarah Lewis, Victor Garcia Satorras, Bastiaan S. Veeling, Frank Noé, Regina Barzilay, Tommi S. Jaakkola

**Affiliations:** Computer Science and Artificial Intelligence Laboratory, Massachusetts Institute of Technology; Department of Statistics, University of Oxford; Department of Engineering, University of Cambridge; Microsoft Research AI4Science; Microsoft Research AI4Science; Microsoft Research AI4Science; Microsoft Research AI4Science; Microsoft Research AI4Science; Microsoft Research AI4Science; Microsoft Research AI4Science; Computer Science and Articial Intelligence Laboratory, Massachusetts Institute of Technology; Computer Science and Articial Intelligence Laboratory, Massachusetts Institute of Technology

## Abstract

Protein design often begins with knowledge of a desired function from a motif which motif-scaffolding aims to construct a functional protein around. Recently, generative models have achieved breakthrough success in designing scaffolds for a diverse range of motifs. However, the generated scaffolds tend to lack structural diversity, which can hinder success in wet-lab validation. In this work, we extend FrameFlow, an SE(3) flow matching model for protein backbone generation, to perform motif-scaffolding with two complementary approaches. The first is *motif amortization*, in which FrameFlow is trained with the motif as input using a data augmentation strategy. The second is *motif guidance*, which performs scaffolding using an estimate of the conditional score from FrameFlow, and requires no additional training. Both approaches achieve an equivalent or higher success rate than previous state-of-the-art methods, with 2.5 times more structurally diverse scaffolds. Code: https://github.com/microsoft/frame-flow.

## 1 Introduction

A common task in protein design is to create proteins with functional properties conferred through a pre-specified arrangement of residues known as a *motif*. The problem is to design the remainder of the protein, called the *scaffold*, that harbors the motif. Motif-scaffolding is widely used, with applications to vaccine and enzyme design (Procko et al., 2014; Correia et al., 2014; Jiang et al., 2008; Siegel et al., 2010). For this problem, diffusion models have greatly advanced capabilities in designing scaffolds (Watson et al., 2023; Wu et al., 2023; Trippe et al., 2022; Ingraham et al., 2023). While experimental wet-lab validation is the ultimate test of a successful scaffold, in this work we focus on improved performance under computational validation of scaffolds. This involves stringent designability^1^ criteria which have been found to correlate well with wet-lab success (Wang et al., 2021). The current state-of-the-art, RFdiffusion (Watson et al., 2023), fine-tunes a pre-trained RosettaFold (Baek et al., 2023) architecture with SE(3) diffusion (Yim et al., 2023b) and is able to successfully scaffold the majority of motifs in a recent benchmark.^2^ However, RFdiffusion suffers from low scaffold diversity. Moreover, the large model size and pre-training used in RFdiffusion make it slow to train and difficult to deploy on smaller machines. In this work, we present a lightweight and easy-to-train generative model that provides equivalent or better motif-scaffolding performance.

**Figure 1:**
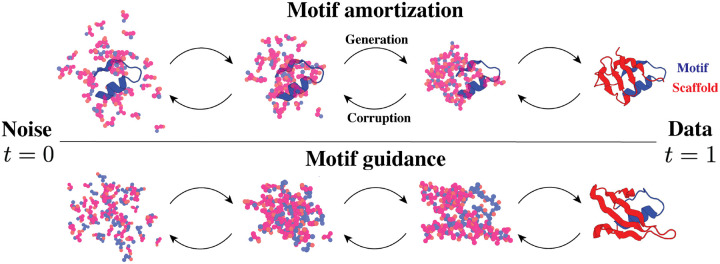
We present two strategies for motif-scaffolding. **Top**: motif amortization trains a flow model to condition on the motif (blue) and generate the scaffold (red). During training, only the scaffold is corrupted with noise. **Bottom**: motif guidance re-purposes a flow model that is trained to generate the full protein for motif-scaffolding. During generation, the motif residues are guided to reconstruct the true motif at t=1 while the flow model will adjust the scaffold trajectory to be consistent with the motif.

To achieve diverse scaffolds without relying on pre-training, we adapt an existing SE(3) flow matching model, FrameFlow (Yim et al., 2023a), for motif-scaffolding. We develop two approaches: (i) *motif amortization*, and (ii) *motif guidance* as illustrated in Fig. 1. Motif amortization was first introduced in RFdiffusion where a *conditional* model is trained to take the motif structure as input when generating the scaffold. We show that FrameFlow can be trained in the same way with improved performance. Motif guidance relies on a Bayesian approach, using an *unconditional* FrameFlow model to sample the scaffold residues, while the motif residues are guided at each step to their final desired end positions. An unconditional model in this context is one that generates the full protein backbone without distinguishing between the motif and scaffold. Motif guidance was described previously in Wu et al. (2023) for SE(3) diffusion. In this work, we develop the extension to SE(3) flow matching.

The two approaches differ fundamentally in whether to train a conditional generative model or to re-purpose an unconditional model for conditional generation. Motif guidance has the advantage that any unconditional model can be used to readily perform motif scaffolding without the need for additional task-specific training. To provide a controlled comparison, we train unconditional and conditional versions of FrameFlow on a dataset of monomers from the Protein Data Bank (PDB) (Berman et al., 2000). Our results provide a clear comparison of the modeling choices made when performing motif-scaffolding with FrameFlow. We find that FrameFlow with both motif amortization and guidance surpasses the performance of RFdiffusion, as measured by the number of unique scaffolds generated^3^ that pass the designability criteria.

Our paper is structured as follows. In Sec. 2 we introduce related work, and in Sec. 3 we provide background on SE(3) flow matching. We present our main contribution extending FrameFlow for motif-scaffolding in Sec. 4. We develop motif amortization for flow matching while motif guidance, originally developed for diffusion models, follows after drawing connections between flow matching and diffusion models. We conclude with results and analysis for motif-scaffolding in Sec. 5. While our work does not introduce novel methodology, we combine existing techniques to develop a motif-scaffolding method that is simple, lightweight, and provides diverse scaffolds. Our contributions are as follows:

- We extend FrameFlow with two fundamentally different approaches for motif-scaffolding: motif amortization and motif guidance. With all other settings kept constant, we show empirical results on motif-scaffolding with each approach.
- Our method can successfully scaffold 21 out of 24 motifs in the motif-scaffolding benchmark compared to 20 with previous state-of-the-art, RFdiffusion, while achieving 2.5 times more unique, designable scaffolds. Our results demonstrate the importance of measuring quality and diversity.

## 2 Related work

### Conditional generation.

The development of conditional generation methods for diffusion and flow models is an active area of research. Two popular diffusion techniques that have been extended to flow matching are classifier-free guidance (CFG) (Dao et al., 2023; Ho & Salimans, 2022; Zheng et al., 2023) and reconstruction guidance (Pokle et al., 2023; Ho et al., 2022; Song et al., 2022; Chung et al., 2022). Motif guidance is an application of reconstruction guidance for motif-scaffolding. Motif amortization is most related to data-dependent couplings (Albergo et al., 2023), where a flow is learned with conditioning of partial data.

### Motif-scaffolding.

Wang et al. (2021) first formulated motif-scaffolding using deep learning. SMCDiff (Trippe et al., 2022) was the first diffusion model for motif-scaffolding using Sequential Monte Carlo (SMC). The Twisted Diffusion Sampler (TDS) (Wu et al., 2023) later improved upon SMCDiff using reconstruction guidance for each particle in SMC. Our application of reconstruction guidance follows from TDS (with one particle), after we derive the equivalent diffusion model to FrameFlow’s flow matching model.

RFdiffusion fine-tunes a pre-trained RosettaFold architecture to become a motif-conditioned diffusion model. We train a FrameFlow model with the same motif-conditioned training and generation techniques in RFdiffusion that we call motif amortization. Compared to RFdiffusion, our method does not rely on expensive pre-training and uses a 3× smaller neural network^4^. Didi et al. (2023) provides a survey of structure-based motif-scaffolding methods while proposing amortized conditioning with Doob’s h-transform. Motif amortization is similar to amortized conditioning but uses the unnoised motif as input. EvoDiff (Alamdari et al., 2023) differs in using sequence-based diffusion model that performs motif-scaffolding with language model-style masked generation. As baselines, we use RFdiffusion and TDS which achieve previous state-of-the-art results on the motif-scaffolding benchmark.

## 3 Preliminaries: SE(3) flow matching for protein backbones

Flow matching (FM) (Lipman et al., 2023) is a simulation-free method for training continuous normalizing flows (CNFs) Chen et al. (2018), a class of deep generative models that generates data by integrating an ordinary differential equation (ODE) over a learned vector field. Recently, flow matching has been extended to Riemannian manifolds Chen & Lipman (2023), which we rely on to model protein backbones via their local frame SE(3) representation. In Sec. 3.1, we give an introduction to Riemannian flow matching. Sec. 3.2 then briefly describes how flow matching is applied to protein backbones using SE(3) flow matching.

### 3.1 Flow matching on Riemannian manifolds

On a manifold ℳ, the CNF $\phi_{t}(\cdot ):ℳ\to ℳ$ is defined via an ordinary differential equation (ODE) along a time-dependent vector field $v(z,t):ℳ\times R\to {𝒯}_{z}ℳ$ where ${𝒯}_{z}ℳ$ is the tangent space of the manifold at z∈ℳ and time is parameterized by t∈[0,1]:

(1) $$\frac{d}{dt}\phi_{t}\left(z_{0}\right)=v\left(\phi_{t}\left(z_{0}\right),t\right)\text{ with boundary condition }\phi_{0}\left(z_{0}\right)=z_{0}\text{.}$$

Starting with $z_{0}\sim p_{0}$ from an easy-to-sample prior distribution, evolving the samples according to Eq. (1) induces a new distribution referred as the push-forward $p_{t}={\left[\phi_{t}\right]}_{*}p_{0}$. One wishes to find a vector field v such that the push-forward $p_{t=1}={\left[\phi_{t=1}\right]}_{*}p_{0}$ (at some arbitrary end time t=1) matches the data distribution $p_{1}$. Such a vector field v is in general not available in closed-form, but can be learned by regressing conditional vector fields $u\left(z_{t},t|z_{0},z_{1}\right)=\frac{d}{dt}z_{t}$ where $z_{t}=\phi_{t}\left(z_{0}|z_{1}\right)$ interpolates between endpoints $z_{0}\sim p_{0}$ and $z_{1}\sim p_{1}$. A natural choice for $z_{t}$ is the geodesic path: $z_{t}={exp}_{z_{0}}\left(t{log}_{z_{0}}\left(z_{1}\right)\right)$, where ${exp}_{z_{0}}$ and ${log}_{z_{0}}$ are the exponential and logarithmic maps at the point $z_{0}$. The conditional vector field takes the following form: $u\left(z_{t},t|z_{1}\right)={log}_{z_{t}}\left(z_{1}\right)/(1-t)$. The key insight of conditional^5^ flow matching (CFM) (Lipman et al., 2023) is that training a neural network $\overset{ˆ}{v}$ to regress the conditional vector field u is equivalent to learning the unconditional vector field v. This corresponds to minimizing the loss function

(2) $$ℒ=E_{t,p_{1}\left(z_{1}\right),p_{0}\left(z_{0}\right)}\left[{\left\|u\left(z_{t},t|z_{1}\right)-\hat{v}\left(z_{t},t\right)\right\|}_{g}^{2}\right].$$

where t~𝒰([0,1]) and $∥\cdot {∥}_{g}^{2}$ is the norm induced by the Riemannian metric g:𝒯ℳ×𝒯ℳ→R. Samples can then be generated by solving the ODE in Eq. (1) using the learned vector field $\overset{ˆ}{v}$ in place of v.

### 3.2 Generative modeling on protein backbones

The atom positions of each residue in a protein backbone can be parameterized by an element T∈SE(3) of the special Euclidean group SE(3) (Jumper et al., 2021; Yim et al., 2023b). We refer to T=(r,x) as a frame consisting of a rotation r∈SO(3) and translation vector $x\in R^{3}$. The protein backbone is made of N residues, meaning it can be parameterized by N frames denoted as $T=\left[T^{\left(1\right)},\ldots ,T^{\left(N\right)}\right]\in SE(3{)}^{N}$. We use bold face to refer to vectors of all the residues, superscripts to refer to residue indices, and subscripts refer to time. Details of the $SE(3{)}^{N}$ backbone parameterization can be found in App. B.1.

We use SE(3) flow matching to develop a generative model of our $SE(3{)}^{N}$ representation of protein backbones. The application of Riemannian flow matching to SE(3) was previously developed in Yim et al. (2023a); Bose et al. (2023). Endowing SE(3) with the product left-invariant metric, the SE(3) manifold effectively behaves as the product manifold $SE(3)=SO(3)\times R^{3}$ (App. D.3 of Yim et al. (2023b)). The vector field over SE(3) can then be decomposed as $v_{SE(3)}^{\left(n\right)}(\cdot ,t)=\left(v_{R}^{\left(n\right)}(\cdot ,t),v_{SO(3)}^{\left(n\right)}(\cdot ,t)\right)$. We parameterize each vector field as

(3) $${\hat{v}}_{\mathbb{R}}^{\left(n\right)}\left({\text{T}}_{t},t\right)=\frac{{\hat{x}}_{1}^{\left(n\right)}\left({\text{T}}_{t}\right)-x_{t}^{\left(n\right)}}{1-t},\;{\hat{v}}_{\text{SO}\left(3\right)}^{\left(n\right)}\left({\text{T}}_{t},t\right)=\frac{{\text{log}}_{r_{t}^{\left(n\right)}}\left({\hat{r}}_{1}^{\left(n\right)}\left({\text{T}}_{t}\right)\right)}{1-t}.$$

The outputs of the neural network are *denoised* predictions ${\overset{ˆ}{x}}_{1}^{\left(n\right)}$ and ${\overset{ˆ}{r}}_{1}^{\left(n\right)}$ which are used to calculate the vector fields. The loss becomes,

(4) $${ℒ}_{\text{SE}\left(3\right)}=E_{t,p_{1}\left({\text{T}}_{1}\right),p_{0}\left({\text{T}}_{0}\right)}\left[{\left\|{\text{u}}_{\text{SE}\left(3\right)}\left({\text{T}}_{t},t|{\text{T}}_{1}\right)-{\hat{\text{v}}}_{\text{SE}\left(3\right)}\left({\text{T}}_{t},t\right)\right\|}_{\text{SE}\left(3\right)}^{2}\right]$$

(5) $$=E_{t,p_{1}\left({\text{T}}_{1}\right),p_{0}\left({\text{T}}_{0}\right)}\left[{\left\|{\text{u}}_{\mathbb{R}}\left({\text{x}}_{t},t|{\text{x}}_{1}\right)-{\hat{\text{v}}}_{\mathbb{R}}\left({\text{T}}_{t},t\right)\right\|}_{\mathbb{R}}^{2}+{\left\|{\text{u}}_{\text{SO}\left(3\right)}\left(\text{r},t|{\text{r}}_{1}\right)-{\hat{\text{v}}}_{\text{SO}\left(3\right)}\left({\text{T}}_{t},t\right)\right\|}_{\text{SO}\left(3\right)}^{2}\right]$$

where we have used bold-face for collections of elements, i.e. $\overset{ˆ}{v}(\cdot )=\left[{\overset{ˆ}{v}}^{\left(1\right)}(\cdot ),\ldots ,{\overset{ˆ}{v}}^{\left(N\right)}(\cdot )\right]$. Our prior is chosen as $p_{0}\left(T_{0}\right)=𝒰(SO(3){)}^{N}⊗\overset{-}{𝒩}{\left(0,I_{3}\right)}^{N}$, where 𝒰(SO(3)) is the uniform distribution over SO(3) and $\overset{-}{𝒩}\left(0,I_{3}\right)$ is the isotropic Gaussian where samples are centered to have zero centre of mass.

Practical details of SE(3) flow matching closely follow FrameFlow (Yim et al., 2023a) where additional auxiliary losses and weights are used. To learn the vector field, we use the neural network architecture from FrameDiff (Yim et al., 2023b) that is comprised of layers that use Invariant Point Attention (IPA) (Jumper et al., 2021) and Transformer blocks (Vaswani et al., 2017). FrameFlow details are provided in App. B.2.

## 4 Motif-scaffolding with FrameFlow

We now describe our two strategies for performing motif-scaffolding with the FrameFlow model: motif amortization (Sec. 4.1) and motif guidance (Sec. 4.2). Recall the full protein backbone is given by $T=\left\{T_{1},T_{2},\ldots ,T_{N}\right\}\in SE(3{)}^{N}$. The residues can be separated into the motif $T^{M}=\left\{T_{i_{1}},\ldots ,T_{i_{k}}\right\}$ of length k where $\left\{i_{1},\ldots ,i_{k}\right\}\subset \{1,\ldots ,N\}$, and the scaffold $T^{S}$ is all the remaining residues, $T=T^{M}\cup T^{S}$. The task of motif-scaffolding can then be framed as a sampling problem of the conditional distribution $p\left(T^{S}|T^{M}\right)$.

### 4.1 Motif amortization

In motif conditioning, we train a variant of FrameFlow that additionally takes the motif as input when generating scaffolds (and keeping the motif fixed). Formally, we model a motif-conditioned CNF,

(6) $$\frac{\text{d}}{\text{d}t}\phi_{t}\left({\text{T}}_{0}^{S}|{\text{T}}^{M}\right)=v\left(\phi_{t},t|{\text{T}}^{M}\right),\;\phi_{0}\left({\text{T}}_{0}^{S}|{\text{T}}^{M}\right)={\text{T}}_{0}^{S}.$$

The flow then transforms a prior density over scaffolds along time as $p_{t}\left(\cdot |T^{M}\right)={\left[\phi_{t}\right]}_{*}p_{0}\left(\cdot |T^{M}\right)$. We use the same prior as in Sec. 3.2, $p_{0}\left(T_{0}^{S}|T^{M}\right)=p_{0}\left(T_{0}^{S}\right)$. We regress to the conditional vector field $u\left(T_{t}^{S},t|T_{1}^{S},T^{M}\right)$ where $T_{t}^{S}$ is defined by interpolating along the geodesic path, $T_{t}^{S}={exp}_{T_{0}^{S}}\left(t{log}_{T_{0}^{S}}\left(T_{1}^{S}\right)\right)$. The implication is that u is conditionally independent of the motif $T^{M}$ given $T_{1}^{S}$. This simplifies our formulation to $u\left(T_{t}^{S},t|T_{1}^{S},T^{M}\right)=u\left(T_{t}^{S},t|T_{1}^{S}\right)$ that is defined in HYPERLINK Sec. 3.2. However, when we learn the vector field, the model needs to condition on $T^{M}$ since the motif placement $T^{M}$ contains information on the true scaffold positions $T_{1}^{S}$. The objective becomes,

(7) $$ℒ=E_{t,p\left({\text{T}}^{M}\right),p_{1}\left({\text{T}}_{1}^{S}|{\text{T}}^{M}\right),p_{0}\left({\text{T}}_{0}^{S}\right)}\left[{\left\|{\text{u}}_{\text{SE}\left(3\right)}\left({\text{T}}_{t}^{S},t|{\text{T}}_{1}^{S}\right)-{\hat{\text{v}}}_{\text{SE}\left(3\right)}\left({\text{T}}_{t}^{S},t|{\text{T}}^{M}\right)\right\|}_{\text{SE}\left(3\right)}^{2}\right].$$

The above expectation requires access to the motif and scaffold distributions, $p\left(T^{M}\right)$ and $p_{1}\left(T_{1}^{S}|T^{M}\right)$, during training. While some labels exist for which residues correspond to the functional motif, the vast majority of protein structures in the PDB do not have labels. We instead utilize unlabeled PDB structures to perform data augmentation (discussed next) that allows sampling a wide range of motifs and scaffolds.

#### Implementation details.

To learn the motif-conditioned vector field ${\overset{ˆ}{v}}_{t}$, we use the FrameFlow architecture with a 1D mask as additional input with a 1 at the location of the motif and 0 elsewhere. To maintain SE(3)-equivariance, it is important to zero-center the motif and initial noise sample from $p_{0}\left(T_{0}^{S}|T^{M}\right)$.

#### 4.1.1 Data augmentation.

**Figure 2:**
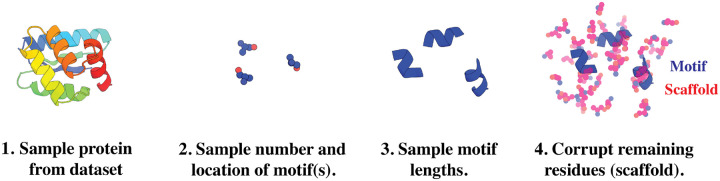
Motif data augmentation. Each protein in the dataset does not come with pre-defined motif-scaffold annotations. Instead, we construct plausible motifs at random to simulate sampling from the distribution of motifs and scaffolds.

Eq. (7) requires sampling from $p\left(T^{M}\right)$ and $p_{1}\left(T_{1}^{S}|T^{M}\right)$, which we do not have access to but can simulate using unlabeled structures from the PDB. Our pseudo-labeled motifs and scaffolds are generated as follows (also depicted in Fig. 2). First, a protein structure is sampled from the PDB dataset. Second, a random number of residues are selected to be the starting locations of each motif. Third, a random number of additional residues are appended onto each motif thereby extending their lengths. Lastly, the remaining residues are treated as the scaffold and corrupted. The motif and scaffold are treated as samples from $p\left(T^{M}\right)$ and $p_{1}\left(T_{1}^{S}|T^{M}\right)$ respectively. Importantly, each protein will be re-used on subsequent epochs where new motifs and scaffolds will be sampled. Our pseudo motif-scaffolds cover a wide range of scenarios that cover multiple motifs of different lengths. We note this strategy was also used in RFdiffusion and bears resemblance to span masking (Hsu et al., 2022). Each step is described in Algorithm 1.


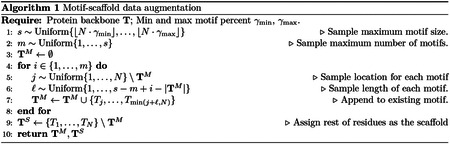


### 4.2 Motif guidance

We now present an alternative Bayesian approach to motif-scaffolding that does not involve learning a motif-conditioned flow model. This can be useful when an unconditional generative flow model is available at hand and additional training is too costly. The idea behind motif guidance, first described as a special case of TDS (Wu et al., 2023) using diffusion models, is to use the desired motif $T^{M}$ to bias the model’s generative trajectory such that the motif residues end up in their known positions. The scaffold residues follow a trajectory that create a consistent whole protein backbone, thus achieving motif-scaffolding.

The key insight comes from connecting flow matching to diffusion models to which motif guidance can be applied. Sampling from the noise distribution $p_{0}$ and then integrating the learned vector field $\overset{ˆ}{v}$ (by minimizing CFM objective in Eq. (5)) allows for sampling $p_{t}\left(x_{t}\right)$

(8) $$\text{d}{\text{T}}_{t}=\hat{\text{v}}\left({\text{T}}_{t},t\right)\text{d}t=\left[f\left({\text{T}}_{t},t\right)-\frac{1}{2}g{\left(t\right)}^{2}\nabla \text{log }p_{t}\left({\text{T}}_{t}\right)\right]\text{d}t,$$

where the RHS is the probability flow ODE (Song et al., 2020), with f and g respectively the drift and diffusion coefficients. Our aim is to sample from the conditional $p\left(T|T^{M}\right)$ from which we can extract $p\left(T^{S}|T^{M}\right)$. We modify Eq. (8) to be conditioned on the motif followed by an application of Bayes rule,

(9) $$\text{d}{\text{T}}_{t}=\left[f\left({\text{T}}_{t},t\right)-\frac{1}{2}g{\left(t\right)}^{2}\nabla \text{log }p_{t}\left({\text{T}}_{t}|{\text{T}}^{M}\right)\right]\text{d}t=\left[f\left({\text{T}}_{t},t\right)-\frac{1}{2}g{\left(t\right)}^{2}\left(\nabla \text{log }p_{t}\left({\text{T}}_{t}\right)+\nabla \text{log }p_{t}\left({\text{T}}^{M}|{\text{T}}_{t}\right)\right)\right]\text{d}t\;=\left[\underset{\text{unconditional vector field }}{\underset{︸}{{\hat{\text{v}}}_{\text{SE}(3)}\left({\text{T}}_{t},t\right)}}-\frac{1}{2}g{\left(t\right)}^{2}\underset{\text{guidance term}}{\underset{︸}{\nabla \text{log }p_{t}\left({\text{T}}^{M}|{\text{T}}_{t}\right)}}\right]\text{d}t.$$

The conditional score in Eq. (9) is unknown, yet it can be approximated by marginalising out $T_{1}$ using the denoised output from the neural network (Song et al., 2022; Chung et al., 2022; Wu et al., 2023),

$$p_{t}\left({\text{T}}^{M}|{\text{T}}_{t}\right)=\int p_{t}\left({\text{T}}^{M}|{\text{T}}_{1}\right)p_{1}\left({\text{T}}_{1}|{\text{T}}_{t}\right)\text{d}{\text{T}}_{1}\approx \int p_{t}\left({\text{T}}^{M}|{\text{T}}_{1}\right)\delta_{{\hat{\text{T}}}_{1}^{M}\left({\text{T}}_{t}\right)}\left({\text{T}}_{t}\right)\text{d}{\text{T}}_{1}=p_{t}\left({\text{T}}^{M}|{\hat{\text{T}}}_{1}^{M}\left({\text{T}}_{t}\right)\right),$$

where $p_{t}\left({\text{T}}^{M}|{\hat{\text{T}}}_{1}^{M}\left({\text{T}}_{t}\right)\right)\propto 𝒩\left({\text{x}}^{M};{\hat{\text{x}}}_{1}^{M}\left({\text{T}}_{t}\right),\omega_{t}^{2}{\text{I}}_{3k}\right)\text{exp}\left(-{\left\|{\text{r}}^{M}-{\hat{\text{r}}}_{1}^{M}\left({\text{T}}_{t}\right)\right\|}_{\text{SO}(3)}^{2}/\omega_{t}^{2}\right)$ is a user-chosen likelihood that quantifies the distance to the desired motif. $\omega_{t}$ is a hyperparameter that controls the magnitude of the guidance which we set to $\omega_{t}^{2}=(1-t{)}^{2}/\left(t^{2}+(1-t{)}^{2}\right)$. We can interpret Eq. (9) as doing unconditional generation by following ${\overset{ˆ}{v}}_{SE(3)}(T,t)$ while $\nabla logp_{t}\left(T^{M}|T_{t}\right)$ guides the motif by minimizing the distance to the true motif. Following SE(3) flow matching, Eq. (9) becomes the following,

(10) $$\text{Translations: d}{\text{x}}_{t}=\left[{\hat{\text{v}}}_{\mathbb{R}}\left({\text{T}}_{t},t\right)+\frac{1}{2}g{\left(t\right)}^{2}\nabla_{{\text{x}}_{t}}{\left\|{\text{x}}^{M}-{\hat{x}}_{1}^{M}\left({\text{T}}_{t}\right)\right\|}_{\mathbb{R}}^{2}/\omega_{t}^{2}\right]\text{d}t,$$

(11) $$\text{Rotations: d}{\text{r}}_{t}=\left[{\hat{\text{v}}}_{\text{SO}(3)}\left({\text{T}}_{t},t\right)+\frac{1}{2}g{\left(t\right)}^{2}\nabla_{{\text{r}}_{t}}{\left\|{\text{r}}^{M}-{\hat{\text{r}}}_{1}^{M}\left({\text{T}}_{t}\right)\right\|}_{\text{SO}(3)}^{2}/\omega_{t}^{2}\right]\text{d}t.$$

We need to choose g(t) such that it matches the learned probability path. This was concurrently done in Pokle et al. (2023), where they showed g(t)=(1-t)/t in the Euclidean setting, see App. C for a derivation. A similar calculation is non-trivial for SO(3), hence we use the same g(t) and observe good performance.

## 5 Experiments

In this section, we report the results of training FrameFlow for motif-scaffolding. Sec. 5.1 describes training, sampling, and metrics. We briefly provide results on unconditional backbone generation in Sec. 5.2. Sec. 5.3 then reports metrics on the motif-scaffolding benchmark introduced in RFdiffusion. Additional motif-scaffolding analysis is provided in App. E.

### 5.1 Set-up

#### Training.

We train two FrameFlow models. FrameFlow-amortization is trained with motif amortization as described in Sec. 4.1 with data augmentation using hyperparameters: $\gamma_{min}=0.05$ so the motif is never degenerately small and $\gamma_{max}=0.5$ to avoid motif being the majority of the backbone. FrameFlow-guidance, to be used in motif guidance, is trained unconditionally on full backbones. Both models are trained using the filtered PDB monomer dataset introduced in FrameDiff. We use the ADAM optimizer (Kingma & Ba, 2014) with learning rate 0.0001. We train each model for 6 days on 2 A6000 NVIDIA GPUs with dynamic batch sizes depending on the length of the proteins in each batch — a technique from FrameDiff.

#### Sampling.

We use the Euler-Maruyama integrator with 500 timesteps for all sampling. We sample 100 scaffolds for each of the 24 monomer motifs following the guidelines in the motif-scaffolding benchmark proposed in RFdiffusion. The benchmark has 25 motifs, but the motif 6VW1 involves multiple chains that FrameFlow cannot handle.

#### Metrics.

Previously, motif-scaffolding was only evaluated through samples passing *designability* (**Des.**). For a description of designability see App. D. In addition to designability, we also calculate the *diversity* (**Div.**) of scaffolds as the number of clusters out of the designable samples. Designing a diverse range of scaffolds increases chances of success in wet-lab validation. This provides an additional data point to check for mode collapse where the model is sampling the same scaffold repeatedly. Clusters are computed using MaxCluster (Herbert & Sternberg, 2008) with TM-score threshold set to 0.5. *Novelty* (**Nov.**) is the average over the TM-score of each sample to its closest protein in the PDB computed using FoldSeek (van Kempen et al., 2022). We only report novelty for unconditional generation as since novelty is not necessarily a desirable property for scaffolds and is expensive for large number of proteins.

### 5.2 Unconditional backbone results

We present backbone generation results of the unconditional FrameFlow model used in FrameFlow-guidance. We do not perform an in-depth analysis since this task is not the focus of our work. Characterizing the backbone generation performance ensures we are using a reliable unconditional model for motif-scaffolding. We evaluate the unconditionally trained FrameFlow model by sampling 100 samples from lengths 70, 100, 200, and 300 as done in RFdiffusion. The results are shown in Tab. 1. We find that FrameFlow achieves slightly worse designability while achieving improved diversity and novelty. We conclude that FrameFlow is able to achieve strong unconditional backbone generation results that are on par with a current state-of-the-art unconditional diffusion model RFdiffusion.

### 5.3 Motif-scaffolding results

**Figure 3:**
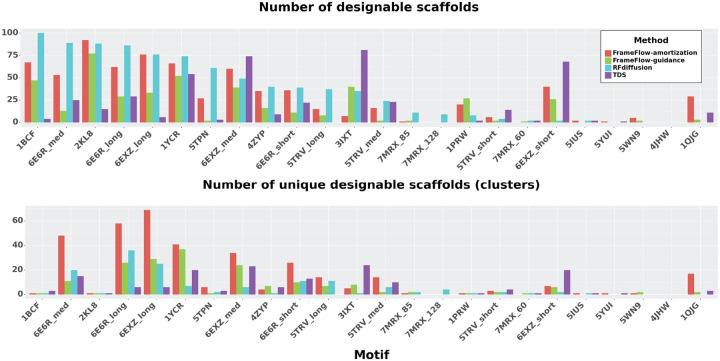
Motif-scaffolding results. (Top) Number of designable scaffolds for each motif using each method. While there is no clear trend, it appears RFdiffusion tends to have the highest success rates. (Bottom) Closer inspection into the number of *unique* designable scaffolds (measured as number of clusters) for each method shows a different story where FrameFlow and TDS have more successful scaffolds.

As baselines, we consider RFdiffusion and the Twisted Diffusion Sampler (TDS). We downloaded RFdiffusion’s published samples and re-ran TDS with k=8 particles — note TDS uses 8 times more neural network evaluations. We refer to FrameFlow-amortization as our results with motif amortization while FrameFlow-guidance uses motif guidance. Fig. 3 shows how each method fares against each other in designability and diversity. In general, RFdiffusion tends to get higher designable success rates, but FrameFlow-amortization and FrameFlow-guidance are able to achieve more unique scaffolds per motif. TDS achieves lower designable scaffolds on average, but demonstrates strong performance on a small subset of motifs.

**Figure 4:**
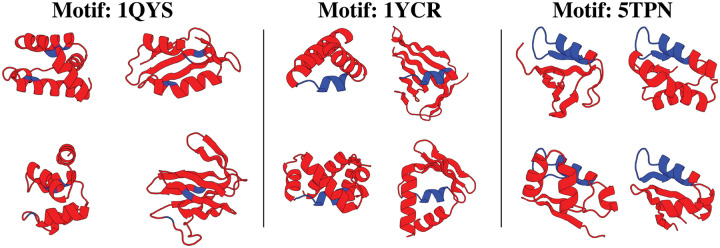
FrameFlow-amortization diversity. In blue is the motif while red is the scaffold. For each motif (1QJG, 1YCR, 5TPN), we show FrameFlow-amortization can generate scaffolds of different lengths and various secondary structure elements for the same motif. Each scaffold is in a unique cluster.

Tab. 2 provides the number of motifs each method solves – which means at least one designable scaffold is sampled – and the number of total designable clusters sampled across all motifs. Here we see FrameFlow-conditioning solves the most motifs and gets nearly double the number of clusters as FrameFlow-guidance. Fig. 4 visualizes several of the clusters for motifs 1QJG, 1YCR, and 5TPN where FrameFlow can generate nearly 6 times more clusters than RFdiffusion. Each scaffold demonstrates a wide range of secondary structure elments and lengths. App. E provides additional analysis into the FrameFlow motif-scaffolding results.

## 6 Discussion

In this work, we present two methods of motif-scaffolding with FrameFlow. These methods can be used with any flow-based model. First, in motif-amortization we adapt the training of FrameFlow to additionally be conditioned on the motif — in effect turning FrameFlow into a conditional generative model. Second, with motif guidance, we use an unconditionally trained FrameFlow for the task of motif-scaffolding though without any additional task-specific training. We evaluated both approaches, FrameFlow-amortization and FrameFlow-guidance, on the motif-scaffolding benchmark from RFdiffusion where we find both methods achieve competitive results with state-of-the-art methods. Moreover, they are able to sample more unique scaffolds and achieve higher diversity. We stress the need to report both success rate and diversity to detect when a model suffers from mode collapse. Lastly, we caveat that all our results and metrics are computational, which may not necessarily transfer to wet-lab success.

### Future directions.

Our results demonstrate a promising avenue for flow-based models in conditional generation for protein design. We have extended FrameFlow for motif-scaffolding; further extentions include binder, enzyme, and symmetric design — all which RFdiffusion can currently achieve. While motif guidance does not outperform motif amortization, it is possible extending TDS to flow matching could close that gap. We make use of a heuristic for Riemannian reconstruction guidance that may be further improved. Despite our progress, there still remains areas of improvement to achieve success in all 25 motifs in the benchmark. One such improvement is to jointly model the sequence to learn useful features about the motif’s amino acids in generating the scaffold.

## Figures and Tables

**Table 1:** Unconditional generation metrics.

Method	Des.(↑)	Div. (↑)	Nov. (↓)
FrameFlow	0.80	**171**	**0.61**
RFdiffusion	**0.87**	156	0.64

**Table 2:** Motif-scaffolding aggregate metrics

Method	Solved (↑)	Div. (↑)
FrameFlow-conditioning	**21**	**353**
FrameFlow-guidance	20	180
RFdiffusion	20	141
TDS	19	161

**Figure 5:**
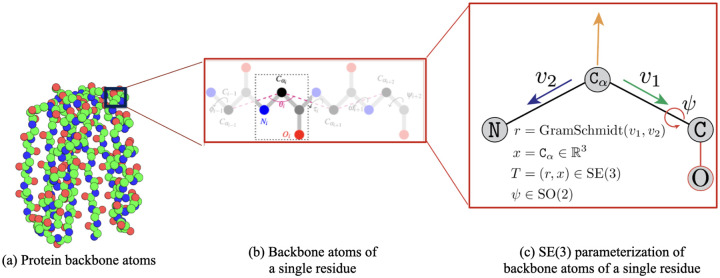
Backbone parameterization with SE(3). (a) Shows the full protein backbone atomic structure without side-chains. (b) Zooms in the backbone atoms of a single residue. Note the repeated arrangements of backbone atoms in each residue. (c) The transformation of turning each set of four backbone atoms into an element of SE(3).

**Figure 6:**
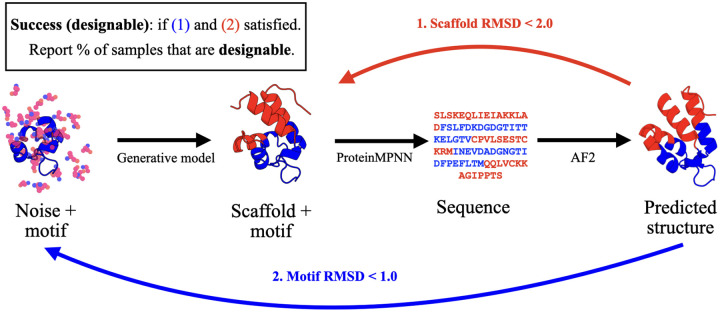
Schematic of computing motif-scaffolding designability. “Generative model” is a stand-in for the method used to generate scaffolds conditioned on the motif. From there, we use ProteinMPNN (Dauparas et al., 2022) to design the sequence then use AlphaFold2 (AF2) (Jumper et al., 2021) in predicting the structure of the sequence. The RMSD is calculated on the scaffold (blue) and motif (red) separately with alignments. A generated scaffold passes designability if the scaffold RMSD < 2.0 and motif RMSD < 1.0.

**Figure 7:**
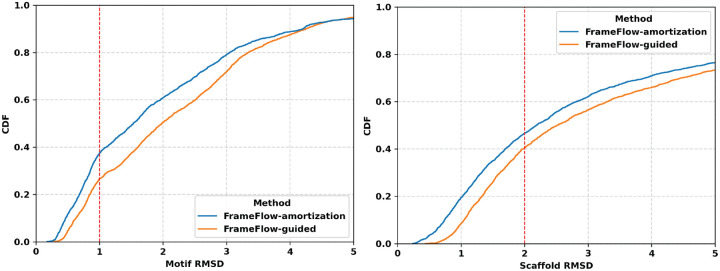
Empirical cumulative density plot of designability RMSD over the motif (left) and scaffold (right) for FrameFlow-amortization (blue line) and FrameFlow-guidance (orange line).

**Figure 8:**
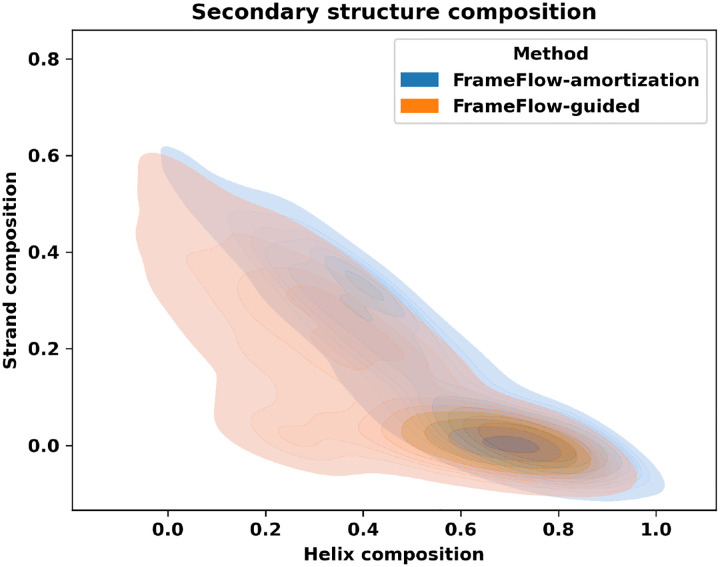
2D kernel density plots of secondary structure composition of *designable* motif-scaffolds from FrameFlow-amortization (blue) and FrameFlow-guided (orange).

**Figure 9:**
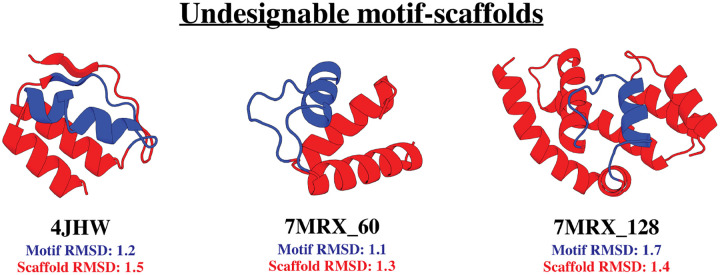
Closest motif-scaffolds from FrameFlow-amortization on the three motifs it fails to solve. We find the > 1.0Åmotif RMSD is the reason for the method failing to pass designability.

**Figure 10:**
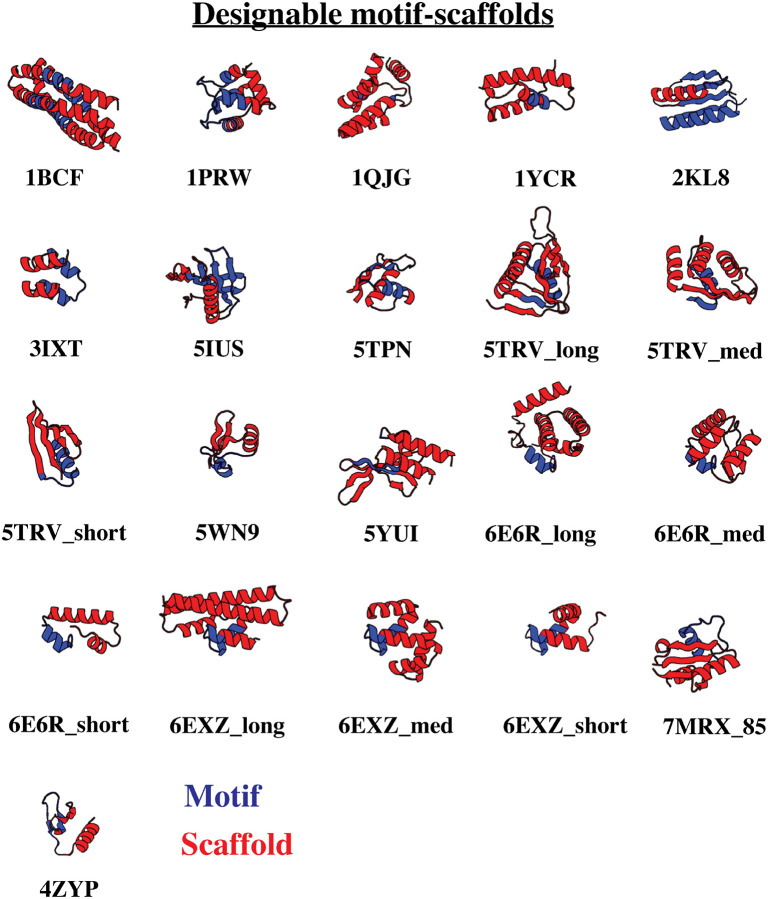
Designable motif-scaffolds from FrameFlow-amortization on 21 out of 24 motifs in the motif-scaffolding benchmark.

## Footnotes

1 A metric based on using ProteinMPNN (Dauparas et al., 2022) and AlphaFold2 (Jumper et al., 2021) to determine the quality of a protein backbone.

2 First introduced in RFdiffusion as a benchmark of 24 single-chain motifs successfully solved across prior works published.

3 The number of unique scaffolds is defined as the number of structural clusters.

4 FrameFlow uses 16.8 million parameters compared to RFdiffusion’s 59.8 million.

5 Unfortunately the meaning of “conditional” is overloaded. The conditionals will be clear from the context.

## Appendix

### A Organisation of appendices

The appendix is organized as follows. App. B provides details and derivations for FrameFlow (Yim et al., 2023a) that we introduce in Sec. 3.2. App. C provides derivation of motif guidance used in Sec. 4.2. Designability is an important metric in our experients, so we provide a description of it in App. D. Lastly, we include additional motif-scaffolding results and analysis in App. E.

## B FrameFlow details

### B.1 Backbone SE(3) representation

A protein can be described by its sequence of residues, each of which takes on a discrete value from a vocabulary of amino acids, as well as the 3D structure based on the positions of atoms within each residue. The 3D structure in each residue can be separated into the backbone and side-chain atoms with the composition of backbone atoms being constant across all residues while the side-chain atoms vary depending on the amino acid assignment. For this reason, FrameFlow and previous SE(3) diffusion models (Watson et al., 2023; Yim et al., 2023b) only model the backbone atoms with the amino acids assumed to be unknown. A second model is typically used to design the amino acids after the backbone is generated. Each residue’s backbone atoms follows a repeated arrangement with limited degrees of freedom due to the rigidity of the covalent bonds. AlphaFold2 (AF2) (Jumper et al., 2021) proposed a SE(3) parameterization of the backbone atoms that we show in Fig. 5. AF2 uses a mapping of four backbone atoms to a single translation and rotation that reduces the degrees of freedom in the modeling. It is this SE(3) representation we use when modeling protein backbones. We refer to Appendix I of Yim et al. (2023b) for algorithmic details of mapping between elements of SE(3) and backbone atoms.

### B.2 SE(3) flow matching implementation

This section provides implementation details for SE(3) flow matching and FrameFlow. As stated in Sec. 3.1, $SE(3{)}^{N}$ can be characterized as the product manifold $SE(3{)}^{N}=R^{N}\times SO(3{)}^{N}$. It follows that flow matching on $SE(3{)}^{N}$ is equivalent to flow matching on $R^{3N}$ and $SO(3{)}^{N}$. We will parameterize backbones with N residues $T=(x,r)\in SE(3{)}^{N}$ by translations $x\in R^{3N}$ and rotations $r\in SO(3{)}^{N}$. As a reminder, we use bold face for vectors of all the residue: $T=\left[T^{\left(1\right)},\ldots ,T^{\left(N\right)}\right],x=\left[x^{\left(1\right)},\ldots ,x^{\left(N\right)}\right],r=\left[r^{\left(1\right)},\ldots ,r^{\left(N\right)}\right]$.

Riemannian flow matching (Sec. 3.1) proceeds by defining the conditional flows,

(12)
$$x_{t}^{\left(n\right)}=\left(1-t\right)x_{0}^{\left(n\right)}+tx_{1}^{\left(n\right)},\;r_{t}^{\left(n\right)}={\text{exp}}_{r_{0}^{\left(n\right)}}\left(t\;{\text{log}}_{r_{0}^{\left(n\right)}}\left(r_{1}^{\left(n\right)}\right)\right),$$

for each residue n∈{1,…,N}. As priors we use $x_{0}^{\left(n\right)}\sim \overset{-}{𝒩}\left(0,I_{3}\right)$ and $r_{0}^{\left(n\right)}\sim 𝒰\left(SO\left(3\right)\right).\;\overset{-}{𝒩}\left(0,I_{3}\right)$ is the isotropic Gaussian in 3D with centering where each sample is centered to have zero mean – this is important for equivariance later on. 𝒰(SO(3)) is the uniform distribution over SO(3). The end points $x_{1}^{\left(n\right)}$ and $r_{1}^{\left(n\right)}$ are samples from the data distribution $p_{1}$.

Eq. (12) uses the geodesic path with linear interpolation; however, alternative conditional flows can be used (Chen & Lipman, 2023). A special property of SO(3) is that ${exp}_{r_{0}}$ and ${log}_{r_{0}}$ can be computed in closed form using the well known Rodrigues’ formula. The corresponding conditional vector fields are

(13)
$$u_{\mathbb{R}}^{\left(n\right)}\left(x_{t}^{\left(n\right)},t|x_{1}^{\left(n\right)}\right)=\frac{x_{1}^{\left(n\right)}-x_{t}^{\left(n\right)}}{1-t},\;u_{\text{SO}\left(3\right)}^{\left(n\right)}\left(r_{t}^{\left(n\right)},t|r_{1}^{\left(n\right)}\right)=\frac{{\text{log}}_{r_{t}^{\left(n\right)}}\left(r_{1}^{\left(n\right)}\right)}{1-t}.$$

We train neural networks to regress the conditional vector fields through the following parameterization,

(14)
$${\hat{v}}_{\mathbb{R}}^{\left(n\right)}\left({\text{T}}_{t},t\right)=\frac{{\hat{x}}_{1}^{\left(n\right)}\left({\text{T}}_{t}\right)-x_{t}^{\left(n\right)}}{1-t},\;{\hat{v}}_{\text{SO}\left(3\right)}^{\left(n\right)}\left({\text{T}}_{t},t\right)=\frac{{\text{log}}_{r_{t}^{\left(n\right)}}\left({\hat{r}}_{1}^{\left(n\right)}\left({\text{T}}_{t}\right)\right)}{1-t},$$

where the neural network outputs the *denoised* predictions ${\overset{ˆ}{x}}_{1}^{\left(n\right)}$ and ${\overset{ˆ}{r}}_{1}^{\left(n\right)}$ while using the noised backbone $T_{t}$ as input. We now modify the loss from Eq. (5) with practical details from FrameFlow,

(15)
$${ℒ}_{\text{SE}\left(3\right)}=E_{t,p_{1}\left({\text{T}}_{1}\right),p_{0}\left({\text{T}}_{0}\right)}\left[{ℒ}_{\mathbb{R}}\left({\text{T}}_{t},{\text{T}}_{1},t\right)+2{ℒ}_{\text{SO}\left(3\right)}\left({\text{T}}_{t},{\text{T}}_{1},t\right)+1(t>0.5){ℒ}_{\text{aux}}\left({\text{T}}_{t},{\text{T}}_{1},t\right)\right]$$

(16)
$${ℒ}_{\mathbb{R}}\left({\text{T}}_{t},{\text{T}}_{1},t\right)={\left\|{\text{u}}_{\mathbb{R}}\left({\text{x}}_{t}|{\text{x}}_{1},t\right)-{\hat{\text{v}}}_{\mathbb{R}}\left({\text{T}}_{t},t\right)\right\|}_{\mathbb{R}}^{2}=\frac{{\left\|{\text{x}}_{1}-{\hat{\text{x}}}_{1}\right\|}_{\mathbb{R}}^{2}}{{\left(1-\text{min}\left(t,0.9\right)\right)}^{2}}$$

(17)
$${ℒ}_{\text{SO}\left(3\right)}\left({\text{T}}_{t},{\text{T}}_{1},t\right)={\left\|{\text{u}}_{\text{SO}\left(3\right)}\left({\text{r}}_{t}|{\text{r}}_{1},t\right)-{\hat{\text{v}}}_{\text{SO}\left(3\right)}\left({\text{T}}_{t},t\right)\right\|}_{\text{SO}\left(3\right)}^{2}=\frac{{\left\|{\text{log}}_{{\text{r}}_{t}^{\left(n\right)}}\left({\text{r}}_{1}^{\left(n\right)}\right)-{\text{log}}_{{\text{r}}_{t}^{\left(n\right)}}\left({\hat{\text{r}}}_{1}^{\left(n\right)}\left({\text{T}}_{t}\right)\right)\right\|}_{\text{SO}\left(3\right)}^{2}}{{\left(1-\text{min}\left(t,0.9\right)\right)}^{2}}.$$

We up weight the SO(3) loss ${ℒ}_{SO(3)}$ such that it is on a similar scale as the translation loss ${ℒ}_{R}$. Eq. (16) is simplified to be a loss directly on the denoised predictions. Both Eq. (16) and Eq. (17) have modified denominators $(1-min(t,0.9){)}^{-2}$ instead of $(1-t{)}^{-1}$ to avoid the loss blowing up near t≈1. In practice, we sample t uniformly from 𝒰[ϵ,1] for small ϵ. Lastly, ${ℒ}_{\text{aux}}$ is taken from section 4.2 in Yim et al. (2023b) where they apply a RMSD loss over the full backbone atom positions and pairwise distances. We found using ${ℒ}_{\text{aux}}$ for all t>0.5 to be helpful. The remainder of this section goes over additional details in FrameFlow.

#### Alternative SO(3) prior.

Rather than using the 𝒰(SO(3)) prior during training, we find using the IGSO3 (σ=1.5) prior (Nikolayev & Savyolov, 1970) used in FrameDiff to result in improved performance. The choice of σ=1.5 will shift the $r_{0}$ samples away from π where near degenerate solutions can arise in the geodesic. During sampling, we still use the 𝒰(SO(3)) prior.

#### Pre-alignment.

Following Klein et al. (2023) and Shaul et al. (2023), we pre-align samples from the prior and the data by using the Kabsch algorithm to align the noise with the data to remove any global rotation that results in a increased kinetic energy of the ODE. Specifically, for translation noise $x_{0}\sim 𝒩{\left(0,I_{3}\right)}^{N}$ and data $x_{1}\sim p_{1}$ where $x_{0},x_{1}\in R^{3\times N}$ we solve $r^{*}=arg{min}_{r\in SO(3)}\;{∥rx_{0}-x_{1}∥}_{R}^{2}$ and use the *aligned* noise $r^{*}x_{0}$ during training. We found pre-aligment to aid in training efficiency.

#### Symmetries.

We perform all modelling within the zero center of mass (CoM) subspace of $R^{N\times 3}$ as in Yim et al. (2023b). This entails simply subtracting the CoM from the prior sample $x_{0}$ and all datapoints $x_{1}$. As $x_{t}$ is a linear interpolation between the noise sample and data, $x_{t}$ will have 0 CoM also. This guarantees that the distribution of sampled frames that the model generates is SE(3)-invariant. To see this, note that the prior distribution is SE(3)-invariant and the learned vector field $v_{SE(3)}$ is equivariant because we use an SE(3)-equivariant architecture. Hence by Köhler et al. (2020), the push-forward of the prior under the flow is invariant.

#### SO(3) inference scheduler.

The conditional flow in Eq. (12) uses a constant linear interpolation along the geodesic path where the distance of the current point x to the endpoint $x_{1}$ is given by a pre-metric $d_{g}:ℳ\times ℳ\to R$ induced by the Riemannian metric g on the manifold. To see this, we first recall the general form of the conditional vector field with $x,x_{1}\in ℳ$ is given as follows (Chen & Lipman, 2023),

(18)
$$u_{t}\left(x|x_{1}\right)=\frac{\text{d log }\kappa \left(t\right)}{\text{d}t}d\left(x,x_{1}\right)\frac{\nabla d\left(x,x_{1}\right)}{{\left\|\nabla d\left(x,x_{1}\right)\right\|}^{2}}$$

(19)
$$=\frac{\text{d log }\kappa \left(t\right)}{\text{d}t}\frac{\nabla d{\left(x,x_{1}\right)}^{2}}{2{\left\|\nabla d\left(x,x_{1}\right)\right\|}^{2}}$$

(20)
$$=\frac{\text{d log }\kappa \left(t\right)}{\text{d}t}\frac{-{\text{log}}_{x}\left(x_{1}\right)}{{\left\|\nabla d\left(x,x_{1}\right)\right\|}^{2}}$$

(21)
$$=\frac{-\text{d log }\kappa \left(t\right)}{\text{d}t}\log_{x}\left(x_{1}\right),$$

with κ(t) a monotonically decreasing differentiable function satisfying κ(0)=1 and κ(1)=0, referred as the *interpolation rate*^6^. Then plugging in a the linear schedule κ(t)=1-t, we recover Eq. (13)

(22)
$$u_{t}\left(x|x_{1}\right)=\frac{-\text{d log }\kappa \left(t\right)}{\text{d}t}{\text{log}}_{x}\left(x_{1}\right)=\frac{1}{1-t}{\text{log}}_{x}\left(x_{1}\right).$$

However, we found this interpolation rate to perform poorly for SO(3) for inference time. Instead, we utilize an exponential scheduler $\kappa (t)=e^{-ct}$ for some constant c. The intuition being that for high c, the rotations accelerate towards the data faster than the translations which evolve according to the linear schedule. The SO(3) conditional flow in Eq. (12) and vector field in Eq. (13) become the following with the exponential schedule,

(23)
$$r_{t}={\text{exp}}_{r_{0}}\left(\left(1-e^{-ct}\right){\text{log}}_{r_{0}}\left(r_{1}\right)\right)$$

(24)
$$v_{r}^{\left(n\right)}=c\;{\text{log}}_{r_{t}^{\left(n\right)}}\left({\hat{r}}_{1}^{\left(n\right)}\right).$$

We find c=10 or 5 to work well and use c=10 in our experiments. Interestingly, we found the best performance when κ(t)=1-t was used for SO(3) during training while $\kappa (t)=e^{-ct}$ is used during inference. We found using $\kappa (t)=e^{-ct}$ during training made training too easy with little learning happening.

The vector field in Eq. (24) matches the vector field in FoldFlow when inference *annealing* is performed (Bose et al., 2023). However, their choice of scaling was attributed to normalizing the predicted vector field rather than the schedule. Indeed they proposed to linearly scale up the learnt vector field via λ(t)=(1-t)c at sampling time, i.e. to simulate the following ODE:

$$\text{d}r_{t}=\lambda \left(t\right)v\left(r_{t},t\right)\text{d}t.$$

However, as hinted at earlier, this is equivalent to using at sampling time a different vector field $\tilde{v}\left(r_{t},t\right)$—induced by an *exponential* schedule $\tilde{\kappa }(t)=e^{-ct}$—instead of the *linear* schedule κ(t)=1-t (that the neural network ${\overset{ˆ}{r}}_{1}^{\theta }$ was trained with). Indeed we have

(25)
$$\tilde{v}\left(r_{t},t\right)=-\partial_{t}\text{ log }\tilde{\kappa }\left(t\right){\text{log}}_{r_{t}}\left({\hat{r}}_{1}\right)=-\frac{-\partial_{t}\text{ log }\tilde{\kappa }\left(t\right)}{-\partial_{t}\text{ log }\kappa \left(t\right)}\partial_{t}\text{ log }\kappa \left(t\right){\text{log}}_{r_{t}}\left({\hat{r}}_{1}\right)$$

(26)
$$=-c\left(1-t\right)\partial_{t}\text{ log }\kappa \left(t\right){\text{log}}_{r_{t}}\left({\hat{r}}_{1}\right)=c\left(1-t\right)v\left(r_{t},t\right)=\lambda \left(t\right)v\left(r_{t},t\right).$$

## C Motif guidance details

For the sake of completeness, we derive in this section the guidance term in Eq. (8) for the flow matching setting. In particular, we want to derive the conditional vector field $v\left(x_{t},t|y\right)$ in terms of the unconditional vector field $v\left(x_{t},t\right)$ and the correction term $\nabla logp_{t}\left(y|x_{t}\right)$. Beware, in the following we adopt the time notation from diffusion models, i.e. t=0 for denoised data to t=1 for fully noised data. We therefore need to swap t→1-t in the end results to revert to the flow matching notations.

Let’s consider the process associated with the following noising stochastic differential equation (SDE)

(27)
$$\text{d}x_{t}=f\left(x_{t},t\right)\text{d}t+g\left(t\right){\text{dB}}_{t}$$

which admits the following time-reversal denoising process

(28)
$$\text{d}x_{t}=\left[f\left(x_{t},t\right)-g{\left(t\right)}^{2}\nabla \text{log }p_{t}\left(x_{t}\right)\right]\text{d}t+g\left(t\right){\text{dB}}_{t}.$$

Thanks to the Fokker-Planck equation, we know that the the following ordinary differential equation admits the same marginal as the SDE Eq. (28):

(29)
$$\text{d}x_{t}=\left[f\left(x_{t},t\right)-\frac{1}{2}g{\left(t\right)}^{2}\nabla \text{log }p_{t}\left(x_{t}\right)\right]\text{d}t\;=v\left(x_{t},t\right)\text{d}t.$$

with $v\left(x_{t},t\right)$ being the probability flow vector field.

Now, conditioning on some observation y, we have

(30)
$$\text{d}x_{t}=v\left(x_{t},t|y\right)\text{d}t\;=\left[f\left(x_{t},t\right)-\frac{1}{2}g{\left(t\right)}^{2}\nabla \text{log }p_{t}\left(x_{t}|y\right)\right]\text{d}t\;=\left[f\left(x_{t},t\right)-\frac{1}{2}g{\left(t\right)}^{2}\left(\nabla \text{log }p_{t}\left(x_{t}\right)+\nabla \text{log }p_{t}\left(y|x_{t}\right)\right)\right]\text{d}t\;=\left[v\left(x_{t},t\right)-\frac{1}{2}g{\left(t\right)}^{2}\nabla \text{log }p_{t}\left(y|x_{t}\right)\right]\text{d}t.$$

As we see from Eq. (30), we only need to know g(t) to adapt reconstruction guidance-which estimates $\nabla logp_{t}\left(y|x_{t}\right)$-to the flow matching setting where we want to correct the vector field. Given a particular choice of interpolation $x_{t}$ from flow matching, let’s derive the associated g(t).

### Euclidean setting

Assume $x_{0}$ is data and $x_{1}$ is noise, with $x_{1}\sim 𝒩(0,I)$. In Euclidean flow matching, we assume a linear interpolation $x_{t}=(1-t)x_{0}+tx_{1}$. Conditioning on $x_{0}$, we have the following conditional marginal density $p_{t|0}=𝒩\left((1-t)x_{0},t^{2}I\right)$. Meanwhile, let’s derive the marginal density ${\tilde{p}}_{t|0}$ induced by Eq. (27). Assuming a linear drift $f\left(x_{t},t\right)=\mu (t)x_{t}$, we know that ${\tilde{p}}_{t|0}$ is Gaussian. Let’s derive its mean $m_{t}=E\left[x_{t}\right]$ and covariance $\Sigma_{t}=Cov\left[x_{t}\right]$. We have that (Särkkä & Solin, 2019)

(31)
$$\frac{\text{d}}{\text{d}t}m_{t}=E\left[f\left(x_{t},t\right)\right]=\mu \left(t\right)m_{t}.$$

thus

(32)
$$E\left[x_{t}\right]=\text{exp}\left(\int_{0}^{t}\mu \left(s\right)\text{d}s\right)x_{0}.$$

Additionally,

(33)
$$\frac{\text{d}}{\text{d}t}\Sigma_{t}=E\left[f\left(x_{t},t\right){\left(m_{t}-x_{t}\right)}^{⊤}\right]+E\left[f{\left(x_{t},t\right)}^{⊤}\left(m_{t}-x_{t}\right)\right]+g{\left(t\right)}^{2}\text{I}$$

(34)
$$=2\mu \left(t\right)\Sigma_{t}+g{\left(t\right)}^{2}\text{I},$$

Matching ${\tilde{p}}_{t|0}$ and $p_{t|0}$, we get

(35)
$$\text{exp}\left(\int_{0}^{t}\mu \left(s\right)\text{d}s\right)x_{0}=\left(1-t\right)x_{0}$$

(36)
$$\Leftrightarrow \int_{0}^{t}\mu \left(s\right)\text{d}s=\text{ln}\left(1-t\right)$$

(37)
$$\Leftrightarrow \mu \left(t\right)=-\frac{1}{1-t}$$

and

(38)
$$2\mu \left(t\right)t^{2}+g{\left(t\right)}^{2}=2t$$

(39)
$$\Leftrightarrow -2\frac{1}{1-t}t^{2}+g{\left(t\right)}^{2}=2t$$

(40)
$$\Leftrightarrow g{\left(t\right)}^{2}=2t+2\frac{1}{1-t}t^{2}$$

(41)
$$\Leftrightarrow g{\left(t\right)}^{2}=\frac{2t}{1-t}.$$

The equivalent SDE that gives the same marginals is Therefore, the following SDE gives the same conditional marginal as flow matching:

(42)
$$\text{d}x_{t}=\frac{-1}{1-t}x_{t}\text{ d}t+\sqrt{\frac{2t}{1-t}}{\text{ dB}}_{t}.$$

### SO(3) setting

The conditional marginal density ${\tilde{p}}_{t|0}$ induced by Eq. (27) with zero drift $f\left(r_{t},t\right)=0$ is given by the IGSO(3) distribution (Yim et al., 2023b): ${\tilde{p}}_{t|0}=IGSO(3)\left(r_{t},r_{0},t\right)$. We are not aware of a closed form formula for the variance of such a distribution.

On the flow matching side, we assume $r_{0}$ is data and $r_{1}$ is noise, with $r_{1}\sim 𝒰(SO(3))$, and a geodesic interpolation $r_{t}={exp}_{r_{0}}\left(t{log}_{r_{0}}\left(r_{1}\right)\right)$. We posit that the induced conditional marginal $p_{t|0}$ is not an IGSO(3) distribution. As such, it appears non-trivial to derive the required equivalent diffusion coefficient g(t) for SO(3). We therefore use as a heuristic the same g(t) as for $R^{d}$.

## D Designability

We provide details of the designability metric for motif-scaffolding and unconditional backbone generation previously used in prior works (Watson et al., 2023; Wu et al., 2023). The quality of a backbone structure is nuanced and difficult to find a single metric for. One approach that has been proven reliable in protein design is using a highly accurate protein structure prediction network to recapitulate the structure after the sequence is inferred. Prior works (Bennett et al., 2023; Wang et al., 2021) found the best method for filtering backbones to use in wet-lab experiments was the combination of ProteinMPNN (Dauparas et al., 2022) to generate the sequences and AlphaFold2 (AF2) (Jumper et al., 2021) to recapitulate the structure. We choose to use the same procedure in determining the computational success of our backbone samples which we describe next. As always, we caveat these results are computational and may not transfer to wet-lab validation. While ESMFold (Jumper et al., 2021) may be used in place of AF2, we choose to follow the setting of RFdiffusion as close as possible.

We refer to *sampled backbones* as backbones generated from our generative model. Following RFdiffusion, we use ProteinMPNN at temperature 0.1 to generate 8 sequences for each backbone in motif-scaffolding and unconditional backbone generation. In motif-scaffolding, the motif amino acids are kept fixed – Protein-MPNN only generates amino acids for the scaffold. The *predicted backbone* of each sequence is obtained with the fourth model in the five model ensemble used in AF2 with 0 recycling, no relaxation, and no multiple sequence alignment (MSA) – as in the MSA is only populated with the query sequence. Fig. 6 provides a schematic of how we compute designability for motif-scaffolding. A sampled backbone is successful or referred to as designable based on the following criterion depending on the task:

- **Unconditional backbone generation**: successful if the Root Mean Squared Deviation (RMSD) of all the backbone atoms is < 2.0Åafter global alignment of the Carbon alpha positions.
- **Motif-scaffolding**: successful if the RMSD of motif atoms is < 1Åafter alignment on the motif Carbon alpha positions. Additionally, the RMSD of the scaffold atoms must be < 2Åafter alignment on the scaffold Carbon alpha positions.

## E FrameFlow motif-scaffolding analysis

In this section, we provide additional analysis into the motif-scaffolding results in Sec. 5.3. Our focus is on analyzing the motif-scaffolding with FrameFlow: motif amortization and guidance. The first analysis is the empirical cumulative distribution functions (ECDF) of the motif and scaffold RMSD shown in Fig. 7. We find that the main advantage of amortization is in having a higher percent of samples passing the motif RMSD threshold compared to the scaffold RMSD. Amortization has better scaffold RMSD but the gap is smaller than motif RMSD. The ECDF curves are roughly the same for both methods.

The next analysis is on the secondary structure composition of each method in Fig. 8. We show the methods are able to achieve a wide range of helical and strand compositions (computed with DSSP (Kabsch & Sander, 1983)) with a concentration around more helical structures. We only plot the composition from *designable* motif-scaffolds. Interestingly, the guidance approach tends to have more loops than amortization. While amortization has another concentration around mixed beta and strand structures.

Lastly, we visualize samples from FrameFlow-amortization on each motif in the benchmark. As noted in Sec. 5.3, amortization is able to solve 21 out of 24 motifs in the benchmark. In Fig. 9, we visualize the generated scaffolds that are closest to passing designability for the 3 motifs it is unable to solve. We find the failure to be in the motif RMSD being over 1ÅRMSD. However, for 4JHW, 7MRX_60, and 7MRX_128 the motif RMSDs are 1.2, 1.1, and 1.7 respectively. This shows amortization is very close to solve all motifs in the benchmark. In Fig. 10, we show designable scaffolds for each of the 21 motifs that amortization solves. We highlight the diverse range of motifs that can be solved as well as diverse scaffolds.
